# Enhancement of Anti-Dermatitis Potential of Clobetasol Propionate by DHA [Docosahexaenoic Acid] Rich Algal Oil Nanoemulsion Gel

**Published:** 2016

**Authors:** Mohammad Sarfaraz Alam, Mohammad Sajid Ali, Foziyah Zakir, Nawazish Alam, Mohammad Intakhab Alam, Faruque Ahmad, Masoom Raza Siddiqui, Mohammad Daud Ali, Mohammad Salahuddin Ansari, Sarfaraz Ahmad, Maksood Ali

**Affiliations:** a *College of Pharmacy, Jazan University, Jazan, KSA.*; b *SBS College of Pharmacy, Patti, Amritsar, Punjab, India. *; c *Department of Clinical Nutrition, College of Applied Medical Sciences, Jazan University, Jazan, KSA. *; d *Chemistry Department, College of Science, King Saud University, Riyadh, KSA. *; e *Department of Pharmacy, Mohammad Al-Mana college for Health Sciences,Al-Rakkah, Dammam, KSA.*; f *College of Pharmacy, AlDawadmi, Shaqra University, KSA.*

**Keywords:** Algal Oil, Anti-inflammatory study, Clobetasol Propionate, Contact Dermatitis, Nanoemulsion

## Abstract

The aim of the present study was to investigate the potential of nanoemulsion formulation for topical delivery of Clobetasol propionate (CP) using algal oil (containing omega-3 fatty acids) as the oil phase. CP has anti-inflammatory, immunomodulatory and antiproliferative activities. However, its clinical use is restricted to some extent due to its poor permeability across the skin. Algal oil was used as the oil phase and was also exploited for its anti-inﬂammatory effect along with CP in the treatment of inﬂammation associated with dermatitis. Nanoemulsion formulations were prepared by aqueous phase titration method, using algal oil, tween 20, PEG 200 and water as the oil phase, surfactant, co-surfactant and aqueous phase respectively. Furthermore, different formulations were subjected to evaluate for ex-vivo permeation and *in-vivo *anti-inflammatory, irritation and contact dermatitis studies. The optimized nanoemulsion was converted into hydrogel-thickened nanoemulsion system (HTN) using carbopol 971 and had a viscosity of 97.57 ± 0.04 PaS. The optimized formulation had small average diameter (120 nm) with zeta potential of -37.01 mV which indicated good long-term stability. *In-vivo *anti-inﬂammatory activity indicated 84.55% and 41.04% inhibition of inflammation for drug loaded and placebo formulations respectively. The assessment of skin permeation was done by DSC and histopathology studies which indicated changes in the structure of epidermal membrane of skin. Contact dermatitis reveals that the higher NTPDase activity in the treatment with the CP-loaded nanoemulsion could be related to the higher anti-inflammatory effect in comparison with placebo nanoemulsion gel.

## Introduction

Contact dermatitis is a frequent inflammatory condition of the skin resulting from exposure to allergens or irritants. Only the superficial regions of the skin *i.e*., epidermis and dermis are affected (1-3). It can cause red, itchy and scaly skin and sometimes the skin becomes blistered, dry and cracked. It is more common in women than men. It is experienced by approximately 1 in 5 women on their hands at some point during their lives (4). The symptomatic treatment of contact dermatitis involves the use of topical corticosteroids particularly because of their vasoconstrictive, anti-inflammatory, immunosuppressive and antiproliferative effects (5). Among the various corticosteroids, Clobetasol propionate (CP) is the drug of choice for the reason that it is highly potent (6). It exerts its action by inhibition of phospholipase A_2_ which leads to the inhibition of synthesis of arachidonic acid and controls the biosynthesis of prostaglandins and leukotrienes (7). However, there are few drawbacks associated with the use of topical CP such as skin atrophy, steroid acne, telangiectasia, hypopigmentation and poor absorption through skin (8). The main limitation lies in the barrier function of the skin, which is considered as one of the most impermeable epithelia of the human body to exogenous substances. Therefore, the major challenges for a topical formulation are to provide a sufficient increase in drug penetration into the skin, without inducing any signiﬁcant irreversible alteration to the skin barrier function (9-11). There has been increased interest during recent years in the use of topical vehicle systems that could modify drug permeation through the skin. Many of the dermal vehicles contain chemical enhancers and solvents to achieve these goals. But use of these chemical enhancers may be harmful, especially in chronic application, as many of them are irritants. Therefore, it is desirable to develop a topical vehicle system that does not require the use of chemical enhancers to facilitate drug permeation through the skin.

Although several attempts have been made to alleviate the adverse effects and improve the therapeutic efficacy. Various colloidal carriers such as poly(d, l-lactic-co-glycolicacid) (PLGA) microspheres, solid lipid nanoparticles, lipid nanospheres, nanostructured lipid carriers, polymeric nanocapsules and lecithin/chitosan nanoparticles containing CP have been developed to enhance the therapeutic efficacy.

Recent novel nanotechnology based delivery systems offer unique properties and substantially improves applications in topical delivery. Nanoemulsions are submicron oil-in-water (o/w) emulsions with mean droplet diameters ranging from 50 to 500 nm (12). Studies have proved that smaller particle size in nanoemulsions ensure close contact with stratum corneum thereby improving the absorption and therapeutic concentration of poorly water soluble drug in the target tissue. In addition it ensures low skin irritation, high solubilization capacity for hydrophilic and lipophilic drugs, and high drug-loading capacity for topical delivery (13). Nanoemulsions act as a depot for slow and controlled release and reduce the frequency of drug administration thus diminishing adverse effects. In our previous study, we have successfully prepared o/w nanoemulsion of CP using eucalyptus oil, tween-20 and ethanol as oil, surfactant and co-surfactant respectively (14). Fontana *et al*., (2009) studied the physicochemical and *in-vitro* release characteristics of CP-loaded polymeric nanoparticles (nanocapsules and nanospheres) and CP-loaded nanoemulsion (15). The controlled release characteristic of nanoparticles was found to be better than nanoemulsion. Moreover the photostability of nanoemulsion was better than nanospheres but worse than nanocapsules. The influence of the polymeric wall of CP-loaded lipid-core nanocapsules or nanoemulsion was evaluated in a model of contact dermatitis after topical administration in rats. The nanocapsules exhibited better control of the drug release and provided better *in-vivo* dermatological efficacy (16). The physical and chemical degradation of clobetasol propionate (CP) incorporated in nanoemulsion was evaluated as per ICH guidelines. The changes in different physicochemical characteristics parameters were found to be statistically insignificant (p ≥ 0.05) after three months of storage. CP exhibited enhanced stability in the nanoemulsion formulation (17). The novelty in the present work lies on the use of algal oil which is rich in omega 3-fatty acid that gets incorporated in the cell membrane and competes with arachidonic acid to reduce the formation of inflammatory mediators. Moreover the drug penetration mechanistic approaches in the dipper layer of skin and anti-inflammatory activity on animal model have been explained.

Thus, the main aim of the present study was to evaluate the potential of algal oil which contains DHA and EPA (18) along with CP in the treatment of inflammation observed in dermatitis, in the form of nanoemulsion using algal oil as the oil phase and other non irritating pharmaceutical acceptable ingredients without using penetration enhancers. Algal oil was used as an excipient as well as an active ingredient. The low viscosity of nanoemulsion restrains its clinical application due to inconvenient use, therefore hydrogel-thickened nanoemulsion (HTN) system were formulated with good stability, powerful permeation ability and suitable viscosity for the topical delivery which provided longer contact with skin. The hydrogel in the formulation will enable close proximity of the formulation with the skin facilitating cutaneous absorption of the active moiety. This will lead to accumulation of drug in the skin and prevent leaching of the drug into systemic circulation. The present investigation focused on the preparation and characterization of HTN system with CP, ex-vivo permeation studies, irritation study, *in-vivo *anti-inﬂammatory activity and their nickel induced dermatitis study of the specialized delivery systems. The long-term goal of this work was to develop safe topical CP formulations for clinical use to increase the anti-dermatitis activity.

## Experimental

*Materials*

Clobetasol propionate was obtained as a gift sample from (Mumbai, India). Omega 3 fatty acid enriched Microalgae DHA Oil was obtained as a gift sample from. China. PEG-400, Tween-80, Tween-20 and ethanol were purchased from Merck (Merck, India). Caprylocaproyl macrogol-6 glycerides (Labrasol), diethylene glycol monoethyl ether (Transcutol-P) and Plurol Oleique were obtained as a kind gift sample from Gattefosse (Mumbai, India). All other chemicals were of analytical grade.

*Screening of Excipients*

An important criterion for screening of components for high loading of drug and more stability in nanoemulsions is the solubility/miscibility of drug in oil, surfactant and co-surfactant. For determination of solubility of CP in algal oil, an excess amount of CP was added to each 5-mL capacity stopper vial and mixed using a vortex mixer (Nickel-Electro Ltd., Oldmixon Crescent, UK). The mixture vial was then kept at 37 ± 1°C in an isothermal shaker (Nirmal International, New Delhi, India) for 72 h to get to equilibrium. The equilibrated samples were removed from the shaker and centrifuged at 3000 rpm for 15 minutes. The supernatant was taken and ﬁltered through a 0.45 µm membrane ﬁlter. The concentration of CP was determined in algal oil by UV spectrophotometer (Shimadzu, Kyoto, Japan) at 241 nm. For selection of surfactant and co-surfactant, miscibility of algal oil was done with a number of surfactants like Tween-20, Tween-80, Labrasol and Tween-60 and co-surfactants like ethanol, Transcutol-P, Plurol oleique, PEG-200 and PEG-400, in 1:1 ratio (oil:surfactant/co-surfactant). Observations were done visually for miscibility. The mixtures which were clear/ transparent in a ratio of 1:1 (v/v) were considered for further studies.

*Phase Studies*

On the basis of solubility/miscibility studies, Tween-20 as a surfactant and Transcutol-P as a co-surfactant were selected for the preparation of nanoemulsion of algal oil. Double distilled water was used as an aqueous phase to avoid surface active impurities. Surfactant and co-surfactant were mixed (S_mix_) in different weight ratios (1: 0, 1: 1, 2: 1, 3: 1, 4: 1, 5: 1) with increasing amount of surfactant with respect to co-surfactant. Sixteen different combinations of oil and S_mix_ (1: 9, 1: 8, 1: 7, 1: 6, 1: 5 1: 4, 1: 3.5, 1: 3, 3: 7, 1: 2, 4: 6, 5: 5, 6: 4, 7: 3, 8: 2 and 9: 1) were made so that maximum ratio could be covered for the study to delineate the boundaries of the phases formed precisely in the phase diagrams. For the determination of existing zone of nanoemulsion, pseudo ternary phase diagrams were constructed using aqueous phase titration method. Slow titration with the aqueous phase was done for each weight ratio of oil and S_mix_, and visual observations were made for transparent and easily ﬂowable oil-in-water (o/w) nanoemulsions. The physical state of nanoemulsion was marked on a pseudo three component phase diagram with one axis representing the aqueous phase, second representing oil and the third representing a mixture of surfactant and co-surfactant at ﬁxed weight ratio (S_mix_ ratio) (19).

*Selection of Formulations*

Among the pseudoternary phase diagrams showing maximum nanoemulsion area, a number of formulations were selected covering the entire range of nanoemulsion occurrence in the phase diagrams with minimum surfactant and maximum water concentration (20-21). Exactly 0.05% w/w of CP, which was kept constant in all the selected formulations, was added to the oil phase during the formulation of nanoemulsions. Selected formulations were subjected to various physical stability tests.

*Physical Stability Studies*

To overcome the problem of metastable formulations, physical stability tests were performed. The selected nanoemulsions were subjected to centrifugation at 5000 rpm for 30 min. The formulations that did not show any phase separations were taken for the heating and cooling cycle. Six cycles between refrigerator temperature (4°C) and (45°C) with storage at each temperature of not less than 48 h were done. The formulations which were found stable were subjected to a freeze-thaw cycle test. Formulations were kept in deep freezer (Vestfrost, Delhi, India) at 20°C for 24 h. After 24 h the nanoemulsions were removed and kept at room temperature. The physically stable nanoemulsions returned to their original form within 2-3 minutes, 3 such cycles were repeated (22-23).

*Characterization of Nanoemulsions*

*Particle Size and Zeta Potential*

The average size and polydispersity index of the nanoemulsion droplets were determined by photon correlation spectroscopy (Nano ZS90, Malvern Instrument, U.K.) which is based on the principle of dynamic light scattering. The measurements were performed using a He-Ne laser at 633 nm by using Avalanche photo diode detector. Light scattering was monitored at 25°C at a 90° angle. Droplet size distribution studies were performed at refractive index of 1.40 because the refractive index for all formulation was in this range. The viscosity and dielectric constant of the medium were set at 4.55 mPas and 79.4 respectively. Zeta potential was determined by using second generation PALS (Phase Analysis Light Scattering), called M3PALS which measures the particle velocity.

*Refractive Index, pH and Viscosity*

Viscosity of nanoemulsion was determined by using Brook-ﬁeld DV III ultra V6.0 RV cone and plate rheometer (Brookﬁeld Engineering Laboratories, Middleboro, MA). Refractive index was determined for different nanoemulsion formulations by using Abbe’s refractometer (Nirmal International, Delhi, India) at 25°C in triplicate. The pH was determined for the optimized nanoemulsions by using a calibrated digital pH meter (Mettler Toledo MP 220, Greifensee, Switzerland) in triplicate at room temperature.

*Ex-Vivo Skin Permeation Studies*

Ex-vivo skin permeation studies were performed on a fabricated Franz diffusion cell with an effective diffusional area of 3.14 cm^2^ and 5 ml of receiver chamber capacity using rat abdominal skin. The full-thickness rat skin was excised from the abdominal region, and hairs were removed with an electric clipper. The subcutaneous tissue was removed surgically, and the dermis side was wiped with isopropyl alcohol to remove adhering fat. The cleaned skin was washed with distilled water and stored in the deep freezer at -21°C until further use. The skin was brought to room temperature and mounted between the donor and receiver compartment of the Franz diffusion cell, where the stratum corneum side faced the donor compartment and the dermal side faced the receiver compartment. Initially the donor compartment was empty and the receiver chamber was ﬁlled with acetate buffer pH 5. The receiver ﬂuid was stirred with a magnetic rotor at a speed of 100 rpm, and the assembled apparatus was placed in the oven and the temperature was maintained at 37 ± 1°C. All the receiver ﬂuid was replaced every 30 min to stabilize the skin. It was found that the receiver ﬂuid showed negligible absorbance after 4.5 h and beyond, indicating complete stabilization of the skin. After complete stabilization of the skin, 1 ml of nanoemulsion formulation (0.5 mg/ml CP) was placed into each donor compartment and sealed with paraffin ﬁlm to provide occlusive conditions. Samples were withdrawn at regular intervals (0.5, 1, 2, 3, 4, 5, 6, 7, 8, 9, 10, 12, 20, 22, and 24 h), ﬁltered through a 0.45 µ membrane ﬁlter, and analyzed for drug content by UV spectrophotometer at 241 nm (24).

*Permeation and Distribution Data Analysis*

The cumulative amount of CP permeated through the albino rat skin (Q, g/cm^2^ was plotted as a function of time (t, h) for each formulation. The permeation rate (ﬂux) at the steady state (Jss, g/cm^2^ /h) and lag time were calculated from the slope and intercept of the straight line obtained by plotting the cumulative amount of CP permeated per unit area of skin versus time at steady state condition respectively. Permeability coefficient (Kp was calculated by dividing the ﬂux by initial drug concentration (*C*o) in the donor portion of cell as given below:

Kp = Jss*/C*o

Enhancement ration (*E*r) was calculated by dividing the Jss of the respective formulation by the Jss of the control formulation as given below:

*E*r = Jss of formulation/Jss of control

*Surface Morphology by Transmission Electron Microscopy*

Morphology and structure of the nanoemulsion were studied using Morgagni 268D transmission electron microscopy (TEM) (FEI, Netherland) operating at 70 KV and capable of point to point resolution. Combination of bright ﬁeld imaging at increasing magnification and diffraction modes were used to reveal the form and size of nanoemulsion droplets. In order to perform the TEM observations, a drop of nanoemulsion was applied on carbon coated grid with 2% phosphotungstic acid (PTA) and was left for 30 sec. The dried coated grid was taken on a slide and covered with a cover slip. The slide was observed under the electron microscope.

*Hydrogel-Thickened Nanoemulsion*

The very low viscosity often exhibited by nanoemulsion is inappropriate for topical use. In order to increase the viscosity of nanoemulsion, carbopol-971, carbopol-940, HPMC and sodium alginate were selected as gelling agents. For preparation of nanoemulsion hydrogel, initially 1% w/v concentration of the polymers was slowly mixed with nanoemulsion under stirring until equilibrium was attained. The formed hydrogel were kept for 24 h and checked for physical stability. The clear hydro-gel thickened nanoemulsion was evaluated for viscosity, pH, content uniformity and homogeneity. In order to optimize polymer concentration, hydrogel was prepared with different polymer concentrations (0.5%, 0.8%, and 1%) and evaluated for *in-vitro *permeation study, viscosity and consistency. Content uniformity was carried out to ascertain that concentration of drug in each portion was uniform. For that an accurately weighed quantity of gel (6 g) from 3 different portions was taken and extracted with methanol which was analyzed by using UV spectrophotometer. HTN equivalent to 0.5 mg CP was applied gently on stratum corneum and *in-vitro *permeation study was performed. At the end of the test, the HTN that remained on the skin was removed, cleaned with cotton soaked in a 0.05% sodium lauryl sulphate and washed with distilled water. In order to determine the drug disposition in the skin, it was weighed, cut into small pieces and sonicated for 15 minutes with methanol in order to extract the CP content. The resulting solution was centrifuged and passed through 0.25 *µ*m ﬁlter and drug content (g/mg of skin) was determined spectrophotometerically (25).

*Assessment of Skin Permeation*

The structural and chemical changes in epidermal layer of skin due to the permeation of drug were determined by DSC and histopathological study.

*DSC Studies*

In order to assess mechanism of permeation, thermal transitions in desiccated stratum corneum membranes of rats were investigated using differential scanning calorimetry (DSC). Both treated and untreated skin samples were previously hydrated over a 27% sodium bromide solution for 48 h to ensure 20% hydration. The skin samples were stored in a desiccator, over silica gel, for at least 3 days before thermal analysis. The sheets of skin were cut into small pieces and 4 mg pieces were hermetically sealed in aluminium pans and kept in the DSC unit along with a similar empty pan as a reference. The sample was heated at the rate of 10°C/min from the temperature range of 30-400°C. Nitrogen was used as a purge gas and flow was adjusted to 20 ml/min.

*Histopathology Studies*

Abdominal skin of wistar rats was treated with the optimized CP nanoemulsion gel. After 24 h, the rats were sacriﬁced and skin samples were taken from untreated (control) and treated areas. Each specimen was stored in 10% formalin solution in phosphate buffer saline (pH 7.4). The specimens were cut into sections vertically. Each section was dehydrated using ethanol embedded in paraffin wax for fixing and stained with hematoxylin and eosin. These samples were then observed under light microscope (Motic, Japan) and compared with control samples.

*In-vivo Anti-Inflammatory Study*

The protocol to carry out *in-vitro* permeation studies was approved by the Institutional Animal Ethics Committee S.B.S College of Pharmacy, Patti, Amritsar, Punjab, India.

The committee’s guidelines were followed for the studies. The anti-inﬂammatory and sustaining action of the optimized formulation was evaluated by the carrageenan induced hind paw edema method by using digital plethysmometer (Ugo Basile, Italy) in wistar rats of either sex weighing 180 to 200 g. A left hind paw of each rat was marked, just below tibiotarsal junction, so that every time the paw was dipped up to the fixed mark to ensure constant paw volume. Animals were randomly divided into 3 groups (control, placebo and formulation treated) each containing 6 rats. The placebo formulation contained only algal oil whereas the formulation treated were applied HTN formulations containing algal oil and CP on the dorsal area of 9 cm^2^ gently with the help of micropore adhesive, 0.5 h prior to carrageenan injection. Acute inflammation was produced by injecting 0.1 ml of 1% (w/v) carrageenan suspension in the sub plantar region of the left hind paw 0.5 h after treatment with drug. The paw volume was measured at 0, 1, 2, 3, 6 and 12 h. The amount of paw swelling was determined for 12 h and expressed as percent edema relative to the initial hind paw volume (26). Percent inhibition of edema was calculated for placebo and drug loaded group with respect to control group using the following formula:

Equation 1 $$\%\mathrm{In}h\mathrm{ibition}=\frac{\%\mathrm{Edema}\left(\mathrm{Control}\right)-\%\mathrm{Edema}(\mathrm{Formulation})}{\%\mathrm{Edema}(\mathrm{Control})}$$

*In-vivo Skin irritation test*

 All the materials used for preparation of nanoemulsion fall under generally regarded as safe (GRAS) category. Concentration of all materials is very critical issue for this formulation. Large amount of surfactants is usually irritant to the skin. Therefore skin irritation test was performed to confirm concentration of materials used for nanoemulsion preparation is safe. Van-Abbe *et al*. mentioned that a value between 0 and 9 indicates that the applied formulation is generally non irritant to human skin (1). Skin irritation test was performed using either sex of wistar rats weighing 180–200 g. Wistar rats were divided into 2 groups (n = 6) and applied the following formulations: optimized nanoemulsion and placebo nanoemulsion. The animals were kept under standard laboratory conditions, temperature at 25 ± 1°C and relative humidity (55 ± 5%). The animals were housed in polypropylene cages, six per cage, with free access to standard laboratory diet and water *as *mention above. A single dose of 10 μl of optimized nanoemulsion, Placebo nanoemulsion and marketed cream were applied to the left ear of the rat and the right ear as a control. The development of erythema was monitored for 14 days using the reported method (27).

*Dermatitis induction by 5% nickel sulfate*

Contact dermatitis was induced by 5% nickel sulfate in solid Vaseline similar to the procedure adopted by Brum *et al*. (28-30). Animals (Wister rats) were divided into four sets (n = 8). After tricotomization, all groups received sensitization with nickel sulfate in the abdomen, except the first group which received only solid Vaseline and continued under the same environmental and feeding conditions as the other groups, this being the control group (C). The induction of dermatitis was done 6 days after sensitization by nickel sulfate in solid Vaseline (5 applications with an interval of 72 h) in each ear after tricotomization. The first group which received only solid Vaseline was euthanized 72 h after the last application of the sensitization agent. The second group was induced to allergic contact dermatitis, which was not managed, and the rats were euthanized 72 h after the last application of nickel sulfate, being the positive control (D). The third group (E) received the topical administration in each ear of the placebo nanoemulsion and the fourth (F) received topical administration in each ear of the CP-loaded nanoemulsion. Dose of application for nanoemulsion and marketed formulation was equal to 1 microgram of CP. Group F and G were treated daily with 0.5ml of the drug loaded nanoemulsion and marketed cream for 5 days on days 1, 3 and 5. All formulations were applied uniformly throughout the ear tissue with massage in order to obtain a better drug penetration (29). After the completion of each treatment, the animals were euthanized and the blood was collected by cardiac puncture to determine the NTPDase activity. For NTPDase activity of lymphocytes, mononuclear leukocytes were isolated from rat blood collected with EDTA and separated using Ficoll-Hypaque density gradients as described by Boyum. After the isolation of mononuclear cells, NTPDase activity was determined by colorimetric assay in compliance with Leal *et al*. All samples were run in duplicate or triplicate, and specific activity is reported as nmol Pi released/min/mg protein. Protein was measured by the Coomassie Blue method using bovine serum albumin as the standard as described by Bradford (31-32).

*Statistical Analysis*

Each experiment was conducted in triplicate and data were analyzed using Excel 2007 (Microsoft Ofﬁce, Microsoft Inc., US) and expressed as a mean ± standard deviation (S.D.). Comparison between the differences of means was performed by using paired t-test for paired comparisons where p-values of 0.05 or less were considered signiﬁcant.

## Results and Discussion

*Criteria for Excipient Selection*

The excipients were selected to be nonirritating, nonsensitizing to the skin and pharmaceutically acceptable, and in several cases it should fall into the GRAS (generally regarded as safe) category. Higher solubility of the drug in the oil phase was another important criterion, as it would help the nanoemulsion to maintain the drug in solubilized form. Safety is a major determining factor in choosing a surfactant, as large amounts of surfactant may cause skin irritation. Non-ionic surfactants are considered to be less toxic than ionic surfactants. Another important aspect to be taken into consideration is the selection of surfactants; ideally the hydrophilic lipophilic balance (HLB) value to form the o/w nanoemulsion should be greater than 10. The right blend of low and high HLB surfactants leads to the formation of a stable nanoemulsion formulation. The presence of co-surfactant decreases the bending stress of interface and allows the interfacial ﬁlm sufﬁcient ﬂexibility to take up different curvatures required to form nanoemulsion over a wide range of composition.

*Screening of Excipients*

Drug loading per formulation is a very critical design factor in the development of nanoemulsion systems for poorly soluble drugs, which is dependent on the drug solubility in oil phase. Solubility of CP in algal oil was found to be 19.59 mg/mL which is very good for topical delivery since the dose of CP is very less. But due to the presence of other fatty acid in algal oil, emulsiﬁcation of algal oil is very difﬁcult. For getting good nanoemulsion region in ternary phase diagram, the miscibility of oil with surfactant and co-surfactant is important. Therefore the miscibility of algal oil was performed with different surfactants and co-surfactants (Table 1). Another important criterion is the selection of surfactant with proper HLB value. Hydrophilic surfactant and co-surfactant are considered to prefer the interface and to lower the necessary energy to form the nanoemulsions, consequently improving the stability. For example, the required HLB value to form o/w nanoemulsion is greater than 10. So selection of surfactant and co-surfactant with appropriate HLB value is necessary (8).

The miscibility of algal oil was found to be highest with Tween-20 in case of surfactant and PEG 200 in case of co-surfactant in 1:1 ratio. Apart from this, Tween-20 has high HLB value (13.2) which can provide good emulsiﬁcation to the algal oil. PEG-200 is very good solublizing agent which can provide better penetration to the lipophilic drug such as CP by increasing the solubility of the drug in the lipophilic domain of the stratum corneum. So for the development of pseudoternary phase diagram algal oil was selected as an oil phase, Tween-20 as surfactant and PEG-200 as a co-surfactant.

**Table 1:** Miscibility of algal oil with surfactants and co-surfactants

**Miscibility of algal oil**				
**S. No.**	**With surfactant (1:1)**	**Observation with surfactant**	**With co-surfactant**	**Observation with co-surfactant**
1	Tween 20	Clear	Ethanol	Turbid
2	Tween 80	Turbid	Transcutol P	Turbid
3	Lecithin	Turbid	PEG 200	Clear
4	Unitop 100	Turbid	Pleurol oleique	Turbid

*Phase Studies*

**Figure 1:**
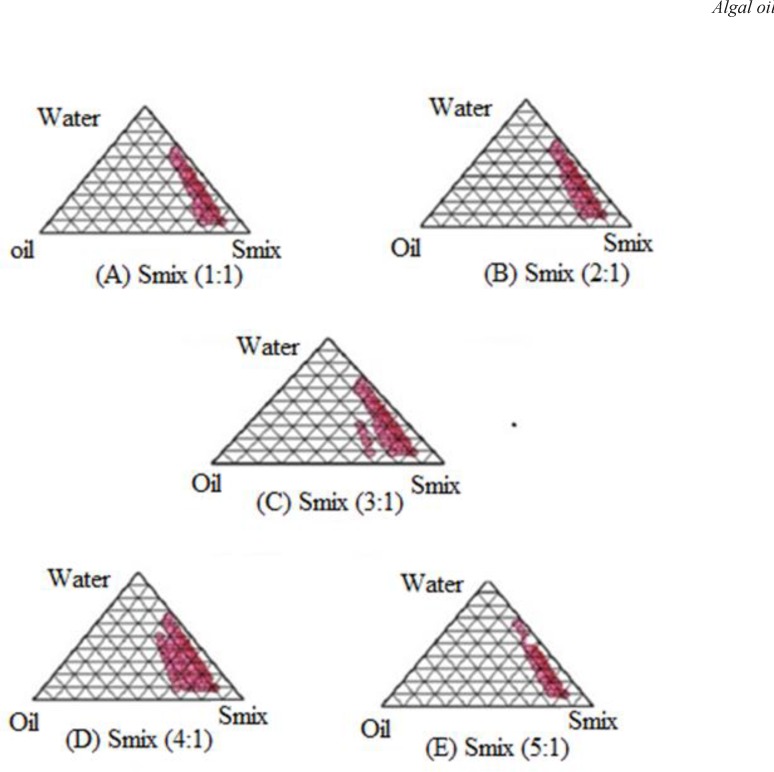
Pseudo-ternary phase diagram for optimizing nanoemulsion formulation

The relationship between the phase behaviour of a mixture and its composition can be captured with the aid of a phase diagram (10). The aim of the construction of pseudo-ternary phase diagram was to ﬁnd out the existence range of nanoemulsion. Care must be taken to ensure that observations are not made on metastable system. Pseudoternary phase diagrams were constructed separately for each *S*_mix_ ratio for getting o/w nanoemulsion regions (Figure 1). The area of nanoemulsion isotropic region changed slightly as the ratio of surfactant in *S*_mix_ was increased. In the phase diagrams, the existence of large or small nanoemulsion region depends on the capability of the particular *S*_mix_ to solubilize the oil phase. The extent of solubilization results in a greater area with the formation of more clear and homogenous solution. In Figure 1, the *S*_mix_ ratio 1:1 (Figure 1A) had a low nanoemulsion area. Thus, oil phase was solubilised to a lesser extent implying that the *S*_mix_ was not able to reduce the interfacial tension of the oil droplets to sufﬁciently low level and thus was not able to reduce the free energy of the system to ultra low level desired to produce nanoemulsions. As the surfactant concentration was increased in *S*_mix_ ratio 2:1 (Figure 1B) a higher nanoemulsion region was observed, perhaps because of further reduction of interfacial tension, increasing the fluidity of interface, thereby increasing entropy of system. As the surfactant concentration was further increased to 3:1 and 4:1 (Figure 1C and D), nanoemulsion region further increased as compared to *S*_mix _ratio 1:1 and 2:1 and was found to be maximum in case of *S*_mix _ratio 4:1. When *S*_mix_ ratio 5:1 was studied, nanoemulsion region decreased slightly as compared to *S*_mix _4:1 which might be due to insufficient amount resulting in the formation of large droplets by diffusion processes driven by the gain in surface free energy. When co-surfactant increases with respect to surfactant as observed in *S*_mix_ (1:2, 1:3, 1:4), a decrease in nanoemulsion region is detected due to insufficient decrease in surface tension. Another rational explanation may be that when temperature quench occurs during stress stability study, instability of nanoemulsion occurs due to separation of oil phase and droplet distribution of smaller size is favoured by the change in curvature free energy (33). Only those formulations, which showed no phase separation, creaming, cracking, coalescence and phase inversion during stress stability tests, were selected for further studies. 

*Selection of Formulation from Phase Diagram*

It is reported that large amount of surfactant causes skin irritation and toxicity related issues therefore it is important to use minimum amount of surfactant and co-surfactant in the formulation. However, for topical delivery, where enhanced skin permeation is the aim, it is not purposeful to select the lowest surfactant concentration. The surfactant concentration should be chosen so that it gives the maximum ﬂux, which is an important criterion but its level should not be toxic to cause any irritation to the skin. This is usually not obtained with formulations that contain the highest amount of surfactant since high surfactant concentration decreases the thermodynamic activity of the drug in the vehicle, and the affinity of the drug to the vehicle becomes greater (5,34,35). While going through pseudoternary phase diagram, oil could be solubilised up to the extent of 40% w/w but in such cases the *S*_mix_ concentration was very high. For the preparation of drug-loaded nanoemulsions, 0.05% CP was dissolved in oil phase. Therefore, from each phase diagram nanoemulsion formulations containing different concentration of oil were selected which contained minimum to maximum *S*_mix_ concentration (Table 2).

**Table 2:** Composition of various CP loaded nanoemulsions

**Formulation Code**	**Algal oil** **(% w/w)**	**S**_mix_ **(% w/w)**	**Distilled water** **(% w/w)**	**CP** **(% w/w)**
A1	7	50	43	0.05
A2	8	46	46	0.05
A3	10	55	35	0.05
B1	5	45	45	0.05
B2	5	40	55	0.05
B3	7	57	36	0.05
B4	9	50	41	0.05
C1	10	51	39	0.05
C2	15	57	28	0.05
C3	20	61	19	0.05

*Physical Stability Studies*

Nanoemulsions are considered to be kinetically stable systems which are formed at a particular concentration of oil, surfactant and water, with no phase separation, creaming or cracking. Selected formulation from phase diagram were subjected to different stress stability testing like heating cooling cycle, centrifugation and freeze thaw cycle. During physical stability testing some formulations became turbid and some showed phase separation. Additionally, when temperature quench occurs during stress stability study, instability of nanoemulsion occurs due to separation of oil phase and droplet distribution of smaller size is favoured by the change in curvature free energy. Formulations with negligible phase separation, creaming, cracking, coalescence and phase inversion during stress stability tests, were selected for further studies (Table 3).

**Table 3.** Physical stability studies of drug loaded formulations

**Formulation Code**	**Heating Cooling Cycles**	**Freeze thaw cycles**	**Centrifugation Studies**
A1	Passed	Failed	Failed
A2	Passed	Passed	Passed
A3	Failed	Failed	Passed
B1	Passed	Passed	Passed
B2	Passed	Passed	Passed
B3	Failed	Passed	Failed
B4	Passed	Passed	Passed
C1	Passed	Passed	Passed
C2	Passed	Passed	Passed
C3	Failed	Failed	Failed

*Characterization of Nanoemulsions*

The formulations which passed physical stability test were evaluated for droplet size, polydispersity index, zeta potential, viscosity, pH, conductivity and refractive index.

*Particle Size and Zeta Potential*

**Figure 2:**
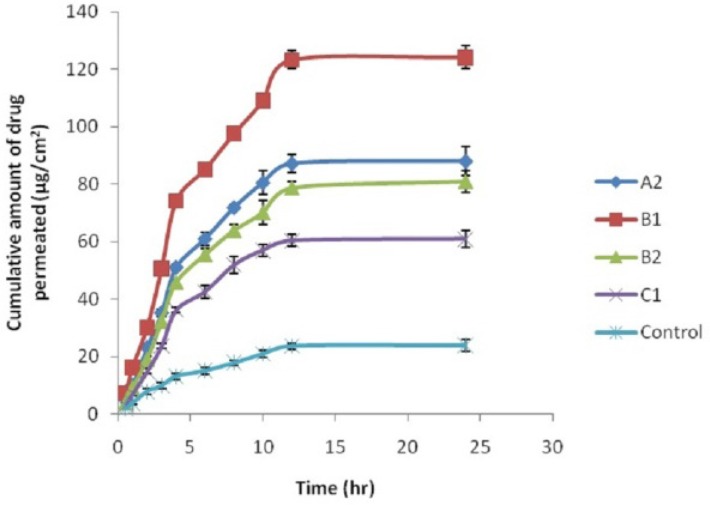
Average droplet size of nanoemulsion formulation B1.

The average droplets size of different nanoemulsions was in the range of 100 to 200 nm. The polydispersity index is a ratio that gives information about the homogeneity of the particle size distribution in a given system. The polydispersity index (Table 4) showed that all the nanoemulsions had narrow size distribution. When the concentration of oil phase was kept constant, it was observed that the decrease in particle size was inversely proportional to the concentration of *S*_mix_. However, the droplet size of all the formulations was in the nano-range. The average particle size and polydispersity index of the formulation B1 was found to be 120 nm (Figure 2) and 0.325 respectively indicating nano-range of droplets with minimum variation in particle size. The zeta potential depends on both the particle surface and the dispersant. Particles interact according to the magnitude of zeta potential and not their surface charge and therefore zeta potential can be used to predict dispersion stability of the system. Zeta potential for formulation B1 was found to be -37.01 mV, which indicated good dispersion stability as it represents signiﬁcant distance between charged particles in dispersion system.

**Table 4:** Evaluation of various drug loaded formulations

**Formulation code**	**Average droplet size (nm) ** **± S.D. (n = 3)**	**Polydispersity index** **± S.D.** **(n = 3)**	**Zeta potential** **(mV)** **± S.D.** **(n = 3)**	**Viscosity (PaS)** **± S.D.** **(n = 3)**	**pH** **± S.D. (n =3)**	**Refractive index** **± S.D.** **(n = 3)**
A2	172 ±2.12	0.671 ±0.03	-24.34 ±1.05	4.55 ±0.03	5.30 ±0.05	1.402 ±0.01
B1	120 ±1.95	0.325 ±0.02	-37.01 ± 0.95	4.42 ±0.02	5.40 ±0.04	1.412 ±0.02
B2	135 ±3.13	0.512 ±0.05	-12.39 ± 0.61	3.91 ±0.03	5.34 ±0.03	1.415 ±0.04
C1	258 ±4.01	0.413 ±0.06	-25.13 ±1.04	4.67 ±0.05	5.27 ±0.04	1.424 ±0.05

*Refractive Index, pH and Viscosity of Nanoemulsion*

Refractive index of a formulation indicates isotropic nature of formulation. Refractive index (Table 4) of nanoemulsion was measured which basically signify the chemical interaction between drug and excipients. There was no signiﬁcant difference in the refractive index value of placebo and drug loaded nanoemulsions so it was concluded that the nanoemulsion formulations were chemically stable and remained isotropic thus showing no interaction between excipient and drug. Acceptable pH of the formulation is another important aspect taken into consideration. Very high or low pH can lead to skin irritation. pH of the formulations were found to be in the range of 5.20-5.44 which are very close to skin pH (pH 4.5–5.5). It was found that viscosity of the formulations depends upon amount of surfactant mixture. As the amount of S_mix_ decreased in a formulation, viscosity also decreased.

*Ex-Vivo Skin Permeation Studies*

**Figure 3:**
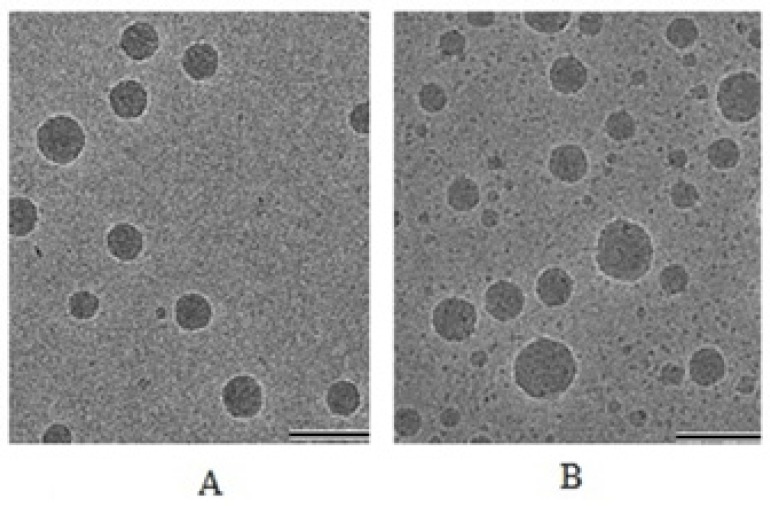
Ex-vivo permeation nanoemulsion study of formulations

The permeation ability of the various nanoemulsions loaded CP and control was evaluated using the ex-vivo permeation experiments. The ex-vivo permeation proﬁles of CP through excised abdominal skins of rat are shown in (Figure 3). A steady increase of CP in the receptor chambers with time was observed. The permeation proﬁles of nanoemulsion were in accordance with the Fick’s diffusion equation. Statistical comparison of the ﬂux throughout 24 h showed that the nanoemulsion preparations of CP provided ﬂux (Table 5) higher than that of the control (CP suspension) which had low cumulative amount of CP at 24 h after application. Cumulative amount of CP permeated from nanoemulsions was 2.55– 5.22 times higher that of the control, 24 h post application. The high permeation rate of nanoemulsions might be attributed to several factors. Firstly, the high concentration of CP released in nanoemulsion resulted in high concentration gradient, which might be the main permeation mechanism of CP into the skin from the formulation. Nanoemulsion could act as drug reservoir where drug is released from the inner phase to outer phase and then further into the skin (36). Secondly, due to the small droplet size, droplets settled down and came in close contact with the skin and a large amount of algal oil in nanoemulsion might have penetrated into the skin. DHA present in algal oil had strong permeation enhancing effect (37). In addition, due to the small droplet diameters of nanoemulsion, the likely mechanism may also be the permeation of CP directly from the droplets into the stratum corneum without nanoemulsion fusion to the stratum corneum and subsequent permeation enhancement. 

**Table 5:** Ex-vivo permeation parameter of CP loaded nanoemulsion formulation and control

**Nanoemulsion**	**Flux Ratio ** **(µg/cm** ^2^ **h**^−1^**)**	**Permeability coefficient (K** **p ** **)**	**Enhancement Rate**
A2	7.27	0.014	3.69
B1	10.27	0.020	5.22
B2	6.56	0.013	3.33
C1	5.03	0.010	2.55
Control	1.96	0.003	1

*Surface Morphology of Particle*

**Figure 4:**
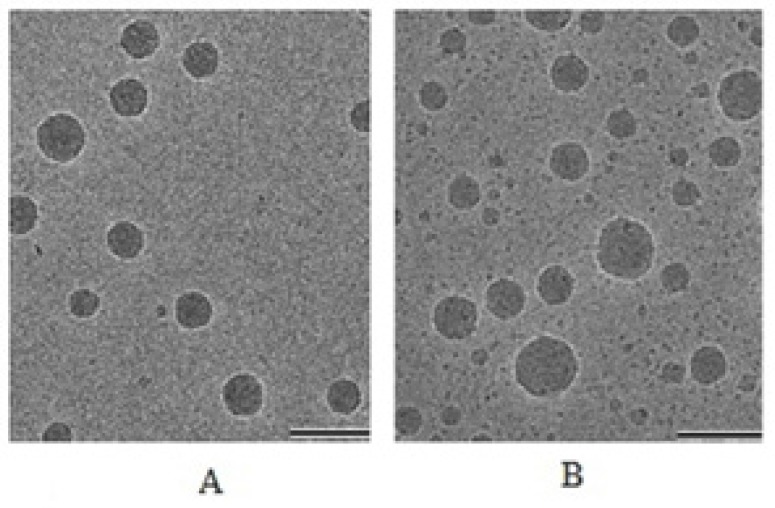
Cryo-electron microscopic images of formulations; A = Placebo; B = Drug loaded nanoemulsion

The TEM studies were carried out to get more insight about the morphology of the nanoemulsion systems. When TEM was performed for optimized formulation it was finally concluded that the particles were spherical in shape and finely distributed within nanometer range. Particle size for placebo and drug loaded formulation was found between 85–110 nm and 96–141 nm (Figure 4) respectively. For CP loaded formulation particle size had increased significantly due to drug entrapment inside the oil droplets. These results were in agreements with the droplet size obtained from photon correlation spectroscopy.

*Hydrogel Thickened Nanoemulsion*

Previously, the gel matrix of nanoemulsion has been prepared with carbomer-940, xanthan gum and sodium alginate for improving the rheological behaviour of nanoemulsion (38-40). In this work, carbopol-971, carbopol-940, HPMC (15 Cps) and sodium alginate were selected at 1% w/v **concentration** for the **preparation of** HTN. However, when carbopol-940 and HPMC were added in nanoemulsion with stirring, only white hydrogel was obtained and the nanoemulsion structure was disturbed. Likewise, when sodium alginate was added in nanoemulsion system it formed hydrogel but after 24 h phase separation occurred. So it was concluded that carbopol-940, sodium alginate and HPMC were not good gel forming polymers for CP loaded nanoemulsion. When carbopol-971 was added in nanoemulsion system a transparent stable hydrogel formed which also maintained nanoemulsion structure of the formulation. As the concentration of polymer was increased its viscosity increased simultaneously. A small quantity of gel was pressed between the thumb and index ﬁnger and the consistency and homogeneity of the gel were observed. The HTN showed absence of any coarse particles. Carbopol-971 in HTN resulted in a high viscosity and oily droplets might be distributed in gel network, which might contribute to the enhancement of the stability of droplets in nanoemulsion. The pH value for all three gel formulations was found in the range 5.15–5.55 (Table 6) which is favourable for topical application. The gels prepared with 0.5 and 0.8% w/v carbopol were not suitable for topical delivery because the consistency of gels was not good. Hydrogel containing 1% carbopol-971 was found to have good viscosity and maximum amount of drug was retained in the skin during permeation study (Table 6). Therefore it was further evaluated for *in-vivo *anti-inﬂammatory study, *in-vivo* irritation study and contact dermetitis.

**Table 6:** Evaluation of hydrogel thickened system

**S. No.**	**Carbopol concentration (%)**	**Viscosity (PaS) ** **± S.D. (n = 3)**	**pH** **± S.D.** **(n = 3)**	**Flux** **(µg/cm** ^2^ **h**^-1^**)**	**Drug retained in skin (µg/mg)**
1	0.4	64.79 ± 0.03	5.15 ± 0.05	8.69	0.25
2	0.8	73.44 ± 0.01	5.43 ± 0.03	5.12	0.34
3	1	97.57 ± 0.04	5.55 ± 0.01	3.93	0.57

*Assessment of Skin Permeation Mechanisms*

*DSC Studies*

**Figure 5:**
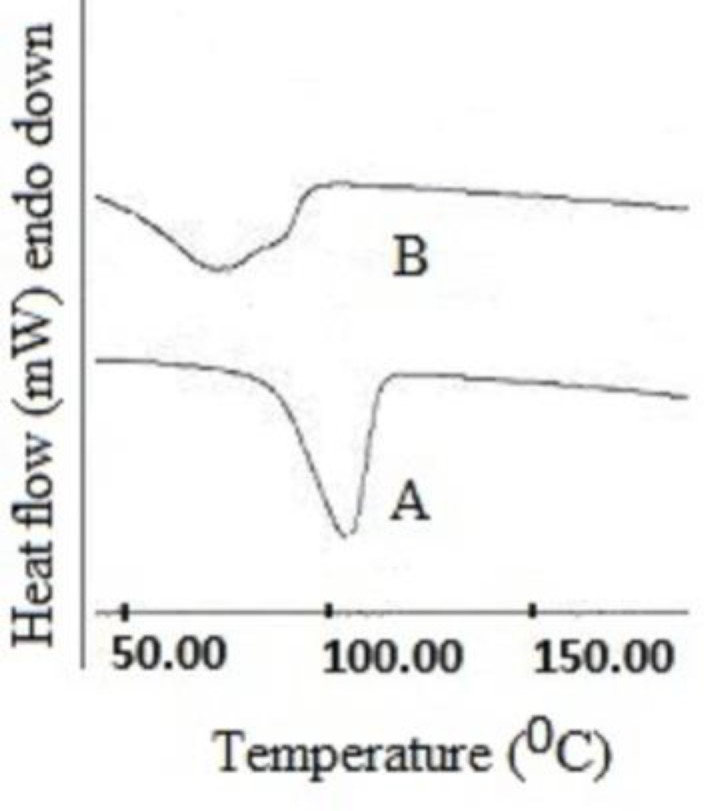
DSC study of rat skin (A) and CP-loaded nanoemulsion treated skin (B).

DSC thermogram showed one prominent peak at 108°C (Figure 5) in untreated skin which was attributed to intracellular keratin denaturation. The treated skin specimen shifted endotherms towards lower melting point 74.55°C. It was observed that nanoemulsion formulations decreased the protein endotherm to lower melting points, suggesting keratin denaturation and possible intracellular permeation mechanism in addition to the extraction of lipid bilayers. One small peak at 32°C was completely removed due to lipid extracting solvent and it completely disappeared in formulation treated skin which indicated that components of nanoemulsions enhanced skin permeation of drugs through extraction of SC lipids (41).

*Histopathology Studies*

**Figure 6:**
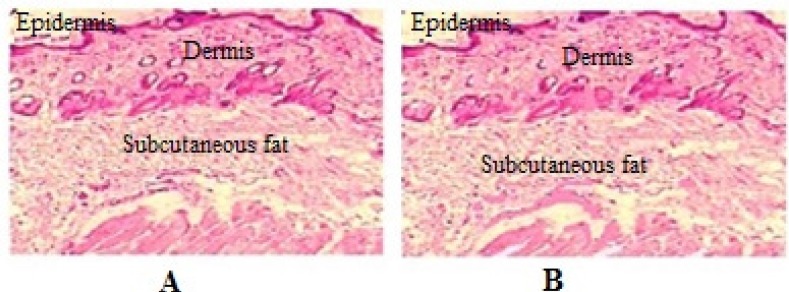
Histological ﬁndings of skin biopsies from wistar rats; control (A), treated with nanoemulsion (B

The inﬂuence of CP loaded nanoemulsion on the anatomical structure of the rat skin is discussed with the aid of light microscopic findings. The photomicrographs of untreated rat skin (control) showed normal skin (Figure 6) with well defined epidermal and dermal layers. Keratin layer was well formed and lied just adjacent to the top most layer of the epidermis. Dermis was devoid of any inﬂammatory cells. When the skin was treated with nanoemulsion formulation, definite changes were observed in the skin morphology. The disruption and extraction of lipid bilayers was clearly evident as distinct voids and empty spaces visible in the epidermal region. The disruption of epidermal layer indicated permeation of CP through stratum corneum (42). The extraction of subcutaneous lipids might cause dehydration of SC leading to signiﬁcant loss of moisture.

*Anti-Inﬂammatory Studies*

The anti-inﬂammatory effects of CP loaded in an optimized HTN were compared with the placebo (algal oil) HTN formulations. The rat’s left footpad became edematous soon after injection of carrageenan and reached its peak at 3 h (76.25%). Signiﬁcant (p < 0.05) amount of % inhibition (84.55%) (Table 7) was achieved in case of CP loaded HTN formulation which indicated good anti-inflammatory activity at the end of 12 h. Besides, 41.04% inhibition was also achieved in placebo (algal oil) HTN formulation at the end of 12 h indicating some anti-inflammatory activity of algal oil which might be due to presence of omega-3 fatty acids. Percentage edema was highest in case of control group and least in case of drug loaded group. This finding revealed synergistic inhibitory effect on inflammation thus suggesting that the optimized formulation could be a promising delivery system for dermatitis treatment. 

**Table 7:** Anti-inflammatory effects of drug loaded and placebo nanoemulsion gel in carrageenan-induced rat paw edema

**Group**	**Formulation**	**N**	**Mean weight ±SD (g)**	**Time (h)**	**% Mean Edema ± SD**	**% Inhibition**
I	Control	6	180.0±12.3	1	29.2±2.3	
	(carrageenan only)			2	43.01±4.2	
				3	76.25±3.3	
				6	59.11±3.7	
				12	39.3±3.01	
II	Drug loaded	6	190±10.01	1	25.5±2.4	12.67
	nanoemulsion hydrogel			2	33.15±2.8	22.92
				3	46.20±4.1	39.40
				6	14.10±1.8	76.16
				12	6.09 ±1.2	84.55
III	Placebo	6	195.0±12.3	1	27.3 ±3.1	6.50
	nanoemulsion hydrogel			2	36.6 ±3.7	14.90
				3	53.19 ±3.4	30.37
				6	36.12 ±3.1	38.89
				12	23.17±2.5	41.04

N = Number of rats in each group; SD = Standard deviation

***Skin irritation test***

The mean values of skin irritation score for drug loaded nanoemulsion gel and placebo nanoemulsion gel were found to be 1.66 ± 0.81 and 0.83±0.75 respectively (Table 8). From these results which were based on 14 days test, it can be concluded that optimized nanoemulsion was safe to be used as topical drug delivery system. It clearly indicated that nanoemulsion has more skin irritation potential due to high amount of surfactant in comparison to placebo nanoemulsion because drug itself may has irritation potential. Overall all the formulation have low irritation score hence it is safe for human use.

**Table 8:** Skin irritation score of the placebo nanoemulsion, drug loaded nanoemulsion and marketed cream

**S. No.**	**Group**	**Score after (day) 1**	**Score after (day) 2**	**Score after (day) 3**	**Score after (day) 4**	**Score after (day) 7**	**Score after (day) 14**	**Mean score± SD**
1	Drug loaded nanoemulsion hydrogel	1	1	3	2	1	2	1.66 ± 0.81
2	Placebo nanoemulsion hydrogel	2	0	2	0	1	2	0.83±0.75

*Contact dermatitis*

**Figure 7:**
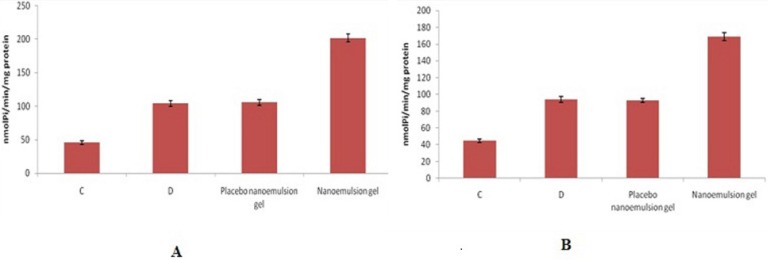
ATP (7a) and ADP (7b) hydrolysis in lymphocytes obtained from the control group (C), contact dermatitis group (D), groups with dermatitis treated with placebo nanoemulsion, and drug loaded nanoemulsion. Data were analyzed statistically by one-way ANOVA followed by the Tukey-Kramer test (a) and Kruskal-Wallis Test (b), (p < 0.05

The *in-vivo *NTPDase activity of lymphocytes after each treatment is shown in (Figure 7). A significant increase in NTPDase activity was observed in lymphocytes of the group treated with CP-loaded nanoemulsion, in relation to ATP and ADP (Figure 7a and 7b, respectively), compared to all other groups (p < 0.05). The ADP and the ATP hydrolysis values for the group treated with CP loaded nanoemulsion and the control groups were not statistically different. The higher NTPDase activity may be associated with the high levels of extracellular ATP resulting from the inflammatory process, which occurs in cases of allergic contact dermatitis. During the dermatitis, these high levels of ATP would have an affinity for P2X7 purinergic receptors, leading to a Th1 pattern of immune response with the production of inflammatory cytokines. The NTPDase would act by decreasing the levels of ATP, which in low concentration would bind to the P2Y receptors, reversing the pattern of immune response to Th2 with the release of anti-inflammatory cytokines. Thus, it is possible that the increased hydrolysis of adenine nucleotides also leads to an increase in the extracellular adenosine concentration, which has immunosuppressive and anti-inflammatory effects. Adenosine plays a central and direct role in the regulation of inflammatory responses and in limiting inflammatory tissue destruction. In this context, the higher NTPDase activity in the treatment with the CP-loaded nanoemulsion, at intervals of 12 h, could be related to the higher anti-inflammatory effect in comparison with placebo nanoemulsion gel. Therefore, the best result observed for the CP-loaded nanoemulsion at 0.05%, even after a longer interval of time, may be related to the slower release of CP. 

## Conclusion

The nanoemulsions containing algal oil and CP were studied for topical delivery. The stable formulations were characterized and converted into transparent hydrogel thickened nanoemulsion for better patient compliance. Drug retention in skin was mechanistically proved by the result of DSC and histopathological study which revealed the disruptions of epidermal layer, responsible for better permeation of drugs. The *in-vivo *anti-inflammatory study showed synergistic effect against inflammation. Contact dermatitis reveals that the higher NTPDase activity in the treatment with the CP-loaded nanoemulsion could be related to the higher anti-inflammatory effect in comparison with placebo nanoemulsion gel. Irritation study confirms that prepared nanoemulsion gel was safe for human use. Therefore it can be concluded that nanocarrier based nanoemulsion hydrogel, containing algal oil and CP might be a good approach for the management of dermatitis.

## Conflict of Interest

The authors state no conﬂict of interest and have received no payment for this project.
